# A small-molecule membrane fluidizer re-sensitizes methicillin-resistant *Staphylococcus aureus* (MRSA) to β-lactam antibiotics

**DOI:** 10.1128/aac.00051-23

**Published:** 2023-09-08

**Authors:** Jessica D. Podoll, Emma Rosen, Wei Wang, Yuefeng Gao, Jing Zhang, Xiang Wang

**Affiliations:** 1 Recreo Pharmaceuticals Inc, Yale Circle, Boulder, Colorado, USA; 2 Department of Chemistry, University of Colorado, Boulder, Colorado, USA; Tufts University-New England Medical Center, Boston, Massachusetts, USA

**Keywords:** antibiotic, antimicrobial resistance, antibiotic adjuvant, membrane fluidizer

## Abstract

Novel antibacterial agents and strategies are urgently needed to fight against the ongoing global antibiotic resistance problem. While natural products remain the main source in antibiotic discovery, synthetic antibacterials provide an attractive alternative and may evade the ancient antibiotic resistance. Herein, we report a small molecule that re-sensitizes methicillin-resistant *Staphylococcus aureus* to β-lactam antibiotics with extremely low potential for resistance development. It belongs to a new class of broad-spectrum antibacterials, trypyricins, which share similar structural characteristics and mechanism of action to the cationic antimicrobial peptides. Mechanistic studies indicated that trypyricins fluidize and disrupt bacterial cytoplasmic membrane. These results suggested that trypyricins represent a promising new class of antibacterials and may be further developed as antibiotic adjuvants to fight against resistant bacteria in the clinic.

## INTRODUCTION

Antibiotic resistance is a major threat to global health and economy (1, 2, 3). Methicillin-resistant *Staphylococcus aureus* (MRSA) is one of the most prevalent resistant bacteria (4). It is resistant to nearly all β-lactam antibiotics due to the expression of the alternative penicillin-binding protein 2a (PBP2a). MRSA infections can be treated with other classes of antibiotics, such as vancomycin. However, vancomycin intermediate-resistant *S. aureus* has become a problem of its own in the clinic. New innovative treatment options, especially those with low potential for resistance development, are urgently needed. A recent survey of the global pre-clinical antibacterial pipeline showed that over 50% of the projects are not traditional direct-acting small molecules, and 70% aim at new targets (5). Despite their high translational risk, it is critical to encourage and further stimulate these innovative approaches to postpone the post-antibiotic era (6).

**FIG 1:**
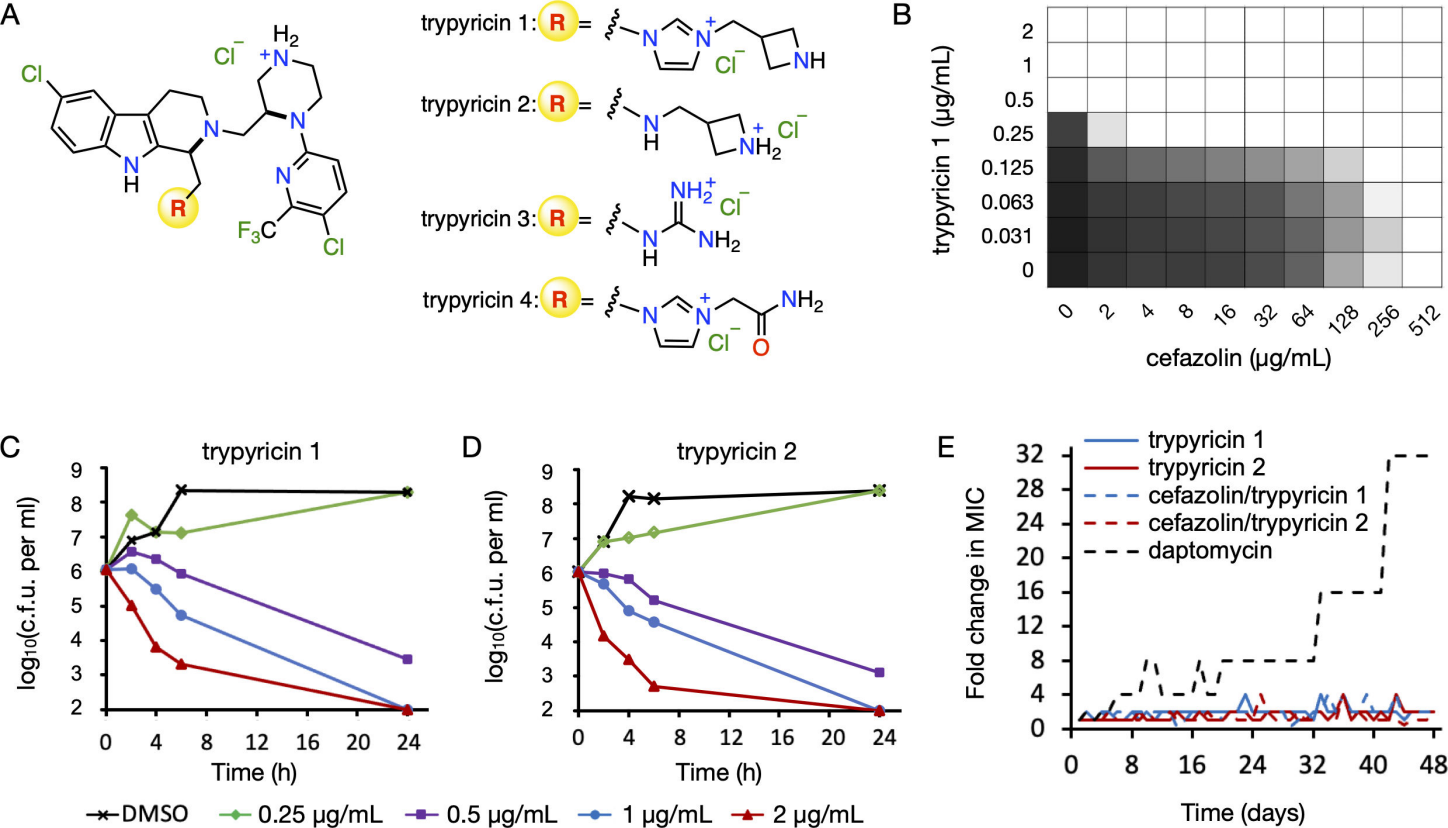
Trypyricins are a new class of antibacterials with low potential for resistance development. (A) Chemical structures of trypyricins 1–4. (B) Trypyricin 1 synergizes with cefazolin in MRSA252. The shade of the well indicates the extent of bacterial growth, and white wells indicate no growth. Time-kill kinetic curves of trypyricin 1 (C) or 2 (D) against *S. aureus* ATCC 29233. (E) Resistance acquisition by serial passaging of MRSA252 in the presence of sub-MIC level of antimicrobials. The *y* axis is the fold of MIC for the passage relative to the MIC of the original strain. This experiment was performed using two biological replicates. ATCC, American Type Culture Collection; MIC, minimum inhibitory concentration.

Antimicrobial peptides (AMPs) are a large family of over 3,000 natural molecules that play an important role in the innate immune system (7). They have attracted extensive interest as therapeutic agents to fight against antibiotic resistance (8, 9). Most AMPs are amphiphilic, possessing both a high proportion of hydrophobic and positively charged residues (10). They are attracted to the negatively charged bacterial membrane, which is distinct from its mammalian counterpart in both composition and charge, and causes bacterial membrane permeabilization and disruption of transmembrane electrochemical gradients or inhibition of cell wall synthesis. In addition, AMPs can also potentiate other classes of antibiotics by permeabilizing bacterial membranes. Colistin has been proven to potentiate carbapenems against a variety of resistant bacteria *in vitro*, *in vivo*, and in the clinic (11). Daptomycin and β-lactams are often used in combination to treat daptomycin-resistant MRSA infections in the clinic (12, 13). However, only seven AMPs have been approved by the U.S. Food and Drug Administration (14). Their development has been hampered by their toxicity, sensitivity to proteases, the difficulty of chemical modifications, and high production costs. Their use in the clinic is also highly limited, either as topical agents or for the treatment of severe infections. Here, we report the discovery of a new class of broad-spectrum antibacterial small molecules, trypyricins (Fig. 1A), with extremely low potential for resistance development. In addition, trypyricin 1 re-sensitizes MRSA to β-lactam antibiotics both *in vitro* and in mice.

## RESULTS

### Antibacterial profiling and mammalian toxicity of trypyricins

Trypyricins are a new class of tryptoline- and pyridine-containing polycationic small molecules. They were developed by systematic optimization of a tricyclic indoline, Of1, that was discovered from bio-inspired diversity synthesis (15). The two aromatic fragments of trypyricins are critical for their ability to potentiate β-lactams, and the polycationic characteristic is responsible for direct bacterial killing activity. Trypyricins showed potent activity against a wide range of Gram-positive and Gram-negative bacteria, including the ESKAPE pathogens (Table 1; SI Appendix, Table S1). Their minimum inhibitory concentrations (MICs) were typically 0.5–1.0 µg/mL against Gram-positive bacteria, such as MRSA, methicillin-susceptible *S. aureus* (MSSA), *Bacillus subtilis* and vancomycin-resistant *Enterococcus faecium* (16). Their MICs were in the range of 2–8 µg/mL against a variety of resistant Gram-negative pathogens, including *Klebsiella pneumoniae*, *Acinetobacter baumannii*, *Pseudomonas aeruginosa*, and *Enterobacter* spp. Unlike cationic AMP, colistin, we found little (twofold) to no changes on the MICs of trypyricins 1 and 2 when the media were supplemented with 50 µg/mL lipopolysaccharide or divalent cations (5 mM of Ca^2+^ or Mg^2+^), respectively. The MICs of trypyricins 1 and 2 increased by twofold and fourfold, respectively, in the presence of human serum albumin, suggesting they have moderate plasma protein-binding capacity. In addition, they exhibited a synergistic effect with β-lactam antibiotics. The fractional inhibitory concentration indices (FICIs) of trypyricins 1 and 2 with cefazolin in MRSA252 were 0.38 (Fig. 1B) and 0.26, respectively (17). Time-dependent killing experiments showed that trypyricins were bactericidal against both Gram-positive *S. aureus* (Fig. 1C and D) and Gram-negative *Escherichia coli* (SI, Fig. S1) with minimum bactericidal concentrations (MBCs) at 1× or 2× of their respective MICs (18). Trypyricin 1 also showed low mammalian toxicity with half inhibitory concentrations (IC_50_s) of 75 and 82 µg/mL in human cell lines HepG2 and HEK293, respectively. In addition, trypyricin 1 did not cause any observable hemolytic activity against human red blood cells at 35 µg/mL, the highest concentration tested. The metabolic stability of trypyricins 1 and 2 was tested in human plasma, and the majority of the compounds, 87% and 97%, respectively, were recovered after 2 h of incubation.

**TABLE 1:** MIC and cytotoxicity of trypyricins 1 and 2 in bacteria and human cell lines

	Concentration (µg/mL)	
Organism and genotype	Trypyricin 1	Trypyricin 2
Bacteria (MIC)^*a*^		
*S. aureus* MRSA252 (MRSA)^*b*^	0.5	0.5
*S. aureus* NRS384 (MRSA)	0.5	0.5
*S. aureus* ATCC 433000 (MRSA)	0.5	0.5
*S. aureus* ATCC BAA-1717 (MRSA)	0.5	0.5
*S. aureus* MRSA252 +4% serum	1	2
*E. faecium* HM-460 (VRE)^*c*^	1	1
*S. aureus* ATCC 29213 (MSSA)^*d*^	0.5	0.5
*B. subtilis* NR-607	0.5	0.5
*E. coli* ATCC 25922	4	2
*K. pneumoniae* ATCC 700603	8	4
*A. baumannii* ATCC BAA-1605	4	2
*P. aeruginosa* ATCC 27853	4	2
*Enterobacter cloacae* ATCC BAA-2468	4	2
Human cell line (IC_50_)		
HepG2	75	19
HEK293	82	32

^*a*^ MICs were determined using cation-adjusted Muller-Hinton broth 2 media. MRSA: methicillin-resistant *S. aureus*.

^*b*^ MRSA, methicillin-resistant *Staphylococcus aureus*.

^*c*^ VRE, vancomycin-resistant *Enterococcus faecium*.

^*d*^ MSSA, methicillin-susceptible S. aureus.

### Resistance studies

**FIG 2:**
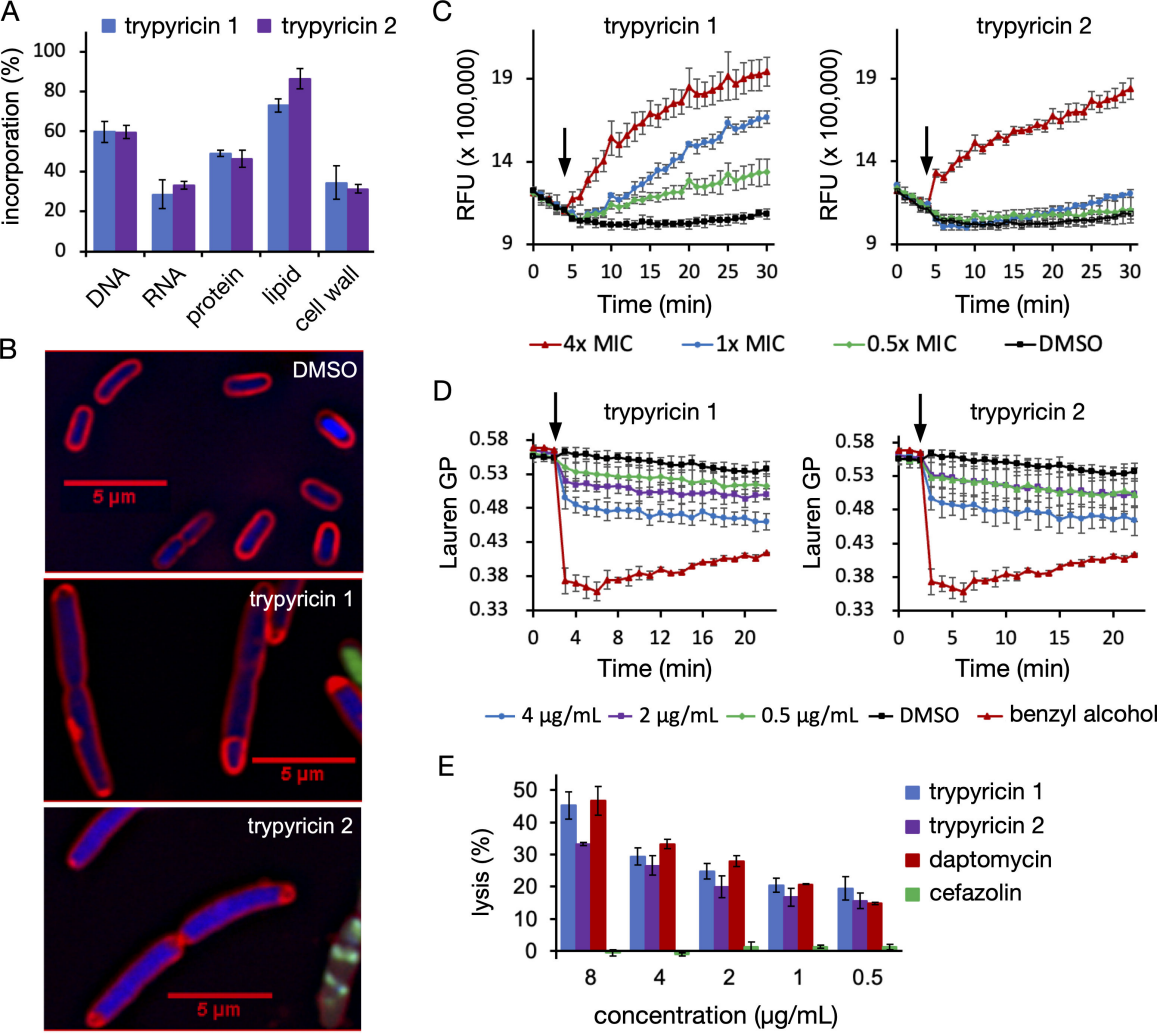
Mechanistic studies of trypyricins. (A) Trypyricins 1 and 2 inhibit all five major biosynthetic pathways in *S. aureus* NRS384 at 2× MIC. (B) Cytological profiling of *E. coli* treated with DMSO, trypyricin 1 or 2 followed by staining with membrane dye FM 4–64 (red), DAPI (blue), and SYTOX Green (green). The rectangular boxes came from adding the scale bar during image processing and could not be removed. (C) Trypyricins induced SYTOX Green uptake in a dose-dependent manner in *B. subtilis*. (D) Trypyricins increased membrane fluidity in *B. subtilis*. (E) trypyricins and daptomycin, but not cefazolin, directly disrupted large unilamellar vesicles with phospholipid compositions resembling the Gram-positive bacterial membrane.

Although trypyricins share more structural features with the cationic AMPs, they exhibited distinct antimicrobial spectra from both the cationic AMP, colistin, and the anionic AMP, daptomycin. These suggested that trypyricins may kill bacteria with a novel mechanism of action. To investigate the mechanism of action, we attempted to select trypyricin-resistant mutants of MRSA252 (19). However, no colonies bearing stable resistance against either trypyricin 1 or 2 were obtained, even when 10^10^ colony-forming units (CFUs) were plated on medium supplemented with 2× or 4× of their respective MICs. We next attempted to generate stable resistance by *in vitro* serial passage of MRSA252 in the presence of sub-MIC of either compound over a period of 48 days (Fig. 2C) (20). For daptomycin, this technique resulted in up to 32-fold increase in MIC. However, only a modest twofold to fourfold increase in MIC was observed against trypyricins, either alone or in combination with cefazolin (1:8 ratio) after 48 days. These results show that the trypyricins have extremely low potential for resistance development, an ideal property for the next-generation antibacterials. Full genomes of the evolved strains were sequenced (Admera Health), and genetic variants were called based on the published MRSA252 sequence (21). Consistent with previous reports, the daptomycin-resistant strain showed a mutation in the *mprF* gene (22). Strains passaged in the presence of trypyricin or trypyricin/cefazolin contained mutations in *mvk*, *accBC*, *capA*, and several others involved in glycerol-phosphate metabolism, including *glpR*, *glpD*, and *glpK* (see full list in SI Appendix*,* Table S2). These genes were implicated in *S. aureus* membrane component biosynthesis, membrane-targeting antibiotic resistance, and the cell envelope stress response, suggesting that trypyricins may target the cell envelope (23 – 26).

### Macromolecular incorporation assay

A macromolecular radiolabel incorporation assay was next performed to determine whether trypyricins 1 and 2 target DNA, RNA, protein, cell wall, or fatty acid biosynthesis (27). All five major biosynthetic pathways were inhibited at 2× and 4× MIC in *S. aureus* (Fig. 2A; SI Appendix, Fig. S2), suggesting that trypyricin treatment resulted in a global energy metabolism failure and a reduction in total biosynthesis, a common profile among antibiotics that target and disrupt the cytoplasmic membrane, such as daptomycin (28, 29).

### Bacterial cytological profiling assay

Cytological profiling of an LPS-deficient *E. coli* strain, CGSC lptD4213, treated with trypyricin was next carried out. Log-phase *E. coli* treated with 5× MIC of trypyricins for 30 min showed membrane phenotypes similar to those produced by membrane-active antibiotics, such as membrane deformations, aberrant septum location, bloated cells, and SYTOX Green uptake (Fig. 2B) (30). We were unable to obtain clear images of *B. subtilis* treated with supra-MIC of trypyricin 1 or 2 due to rapid and significant membrane damage and lysis. However, when treated with sub-MIC of trypyricins, *B. subtilis* cells were found to be bent in certain regions and to possess extra membrane patches and extracellular membrane blebs, and some cells were bloated or elongated at the ends (SI Appendix*,* Fig. S3). These phenotypes are highly similar to those induced by daptomycin, a membrane-targeting AMP. In a separate experiment, we further confirmed that trypyricins induced the uptake of SYTOX Green in both *B. subtilis* and *E. coli* (Fig. 2C; SI Appendix, Fig. S4), suggesting that trypyricins directly disrupt the bacterial cytoplasmic membrane.

### Membrane fluidity assay

Cytoplasmic membrane-targeting antimicrobials often exhibit characteristic effects on global membrane fluidity (31 – 33). Either an increase or a decrease in overall membrane fluidity and organization can change membrane permeability and alter membrane architecture, leading to multiple deleterious effects, including membrane protein mislocalization and breakdown of the proton motive force (34). Examination of bulk membrane properties using the fluorescent indicator Laurdan showed that, when *B. subtilis* was treated with trypyricins, the general polarization (GP) values of Laurdan emission decreased, as observed for a known membrane fluidizer, benzyl alcohol (Fig. 2D) (35). This result indicates that trypyricins caused an increase in membrane fluidity, as opposed to the decrease in membrane fluidity observed in bacteria treated with daptomycin, an anionic AMP (32).

Membrane-targeting antibacterials are desirable because they can destabilize the structural integrity of the cell and disrupt numerous essential cell functions that require a functional cytoplasmic membrane. The β-lactam resistance machinery in MRSA requires functional membrane organization for the β-lactam sensor MecR1 and resistance protein PBP2a for cell wall synthesis in the presence of β-lactam antibiotics (36). MRSA also has been reported to possess a more rigid membrane compared to that of MSSA (37). Therefore, membrane fluidizers may disrupt membrane function and enhance β-lactam activity in MRSA.

### Fluorescence leakage assay using a model membrane

To further confirm that the observed effects of trypyricins on bacterial membranes are not a secondary outcome due to cell death, we tested the interactions of trypyricins with a model membrane. Large unilamellar vesicles (LUVs) with a phospholipid composition resembling that of Gram-positive bacterial membrane (i.e.*,* 50% cardiolipin and 50% phosphoglycerol) were pre-loaded with carboxyfluorescein (38, 39). Like daptomycin, trypyricins disrupted LUVs and caused fluorescein leakage in a concentration-dependent manner (Fig. 2E). These data support the conclusion that trypyricins directly target and disrupt bacterial cytoplasmic membrane.

### *In vivo* studies

We next evaluated the pharmacokinetic (PK) profiles of trypyricins 1 and 2 to determine their suitability for *in vivo* efficacy studies. Mice were administered with 2 mg/kg (mpk) of compound by intravenous (i.v.) injection or 10 mpk via intraperitoneal (i.p.) injection, and the PK parameters were determined. Both trypyricins showed decent bioavailability (63%–64%) and long half-life (>7 h, SI Appendix, Table S3), while trypyricin 1 is superior due to lower clearance and higher exposure.

**FIG 3:**
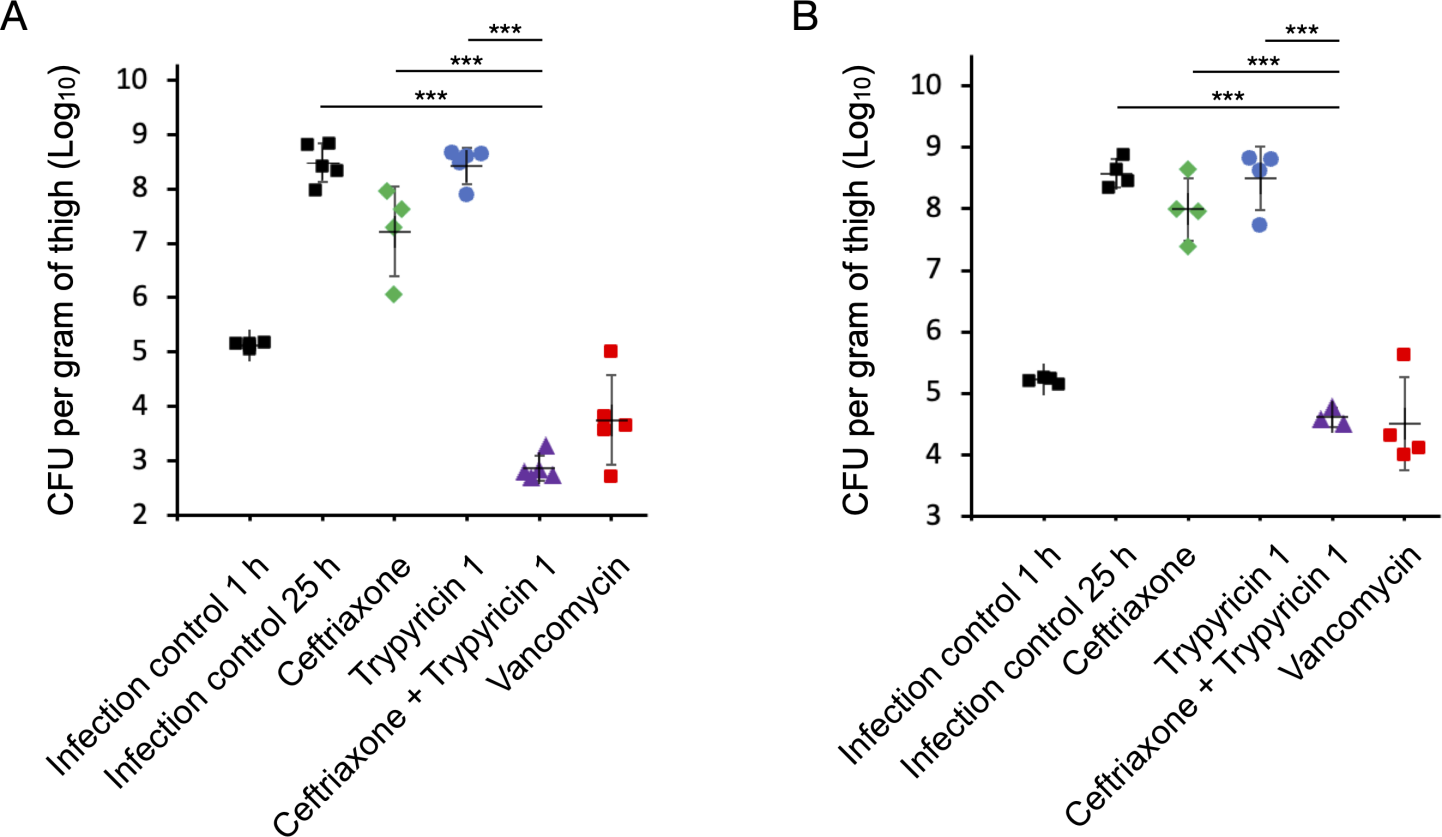
Trypyricin 1 was efficacious in neutropenic mouse thigh infection model with MRSA. Various dose treatments (1 h post-infection) with trypyricin 1 (i.p., 50 mpk, QD), ceftriaxone (s.c., 100 mpk, TID), vancomycin (s.c., 25 mpk, twice a day), or combinations of ceftriaxone (s.c., 100 mpk, TID) and trypyricin 1 (i.p., 50 mpk, QD) in neutropenic mouse thigh model using MRSA (**A**) ATCC 43300 or (**B**) BAA-1717. For controls, CFU in thighs were determined at 1 h and 25 h post-infection. The CFU from each mouse are plotted as individual points and error bars represent the standard deviation within an experimental group. Five animals per group were used in MRSA ATCC 43300 model, and four animals per group were used in MRSA ATCC BAA-1717 model. Data are presented as mean ± SD; ***P < 0.001 (determined using an unpaired, two-tailed *t*-test analysis).

Trypyricin 1 showed excellent *in vitro* activity against MRSA, low cytotoxic and hemolytic activity, and favorable PK profile. Hence, we decided to evaluate its *in vivo* efficacy in a neutropenic mouse thigh infection model using MRSA American Type Culture Collection (ATCC) 43300 (40). A third-generation cephalosporin, ceftriaxone, was selected to study the synergistic effect with trypyricin 1 based on their comparable PK profiles. Three times a day (TID) subcutaneous (s.c.) dosing was chosen for ceftriaxone due to its much shorter half-life of 0.93 h. Trypyricin 1 was dosed at 50 mpk once a day via i.p. injection, alone or in combination with ceftriaxone at 100-mpk TID. The results showed that, on their own, neither trypyricin 1 nor ceftriaxone significantly reduced the bacterial load relative to the untreated control (Fig. 3A). However, their combination resulted in excellent efficacy, with 5.6 log_10_ reduction, which is comparable to the positive control, vancomycin. In addition, trypyricin 1/ceftriaxone also showed excellent efficacy in the same model with another MRSA strain ATCC BAA-1717 (Fig. 3B). In these studies, all dosages were well tolerated by the mice and no adverse effects were observed. Trypyricin 1 did not decrease bacterial load by itself, probably because the high plasma concentration (>30%) in mice decreased the effective concentration of trypyricin 1. However, it was able to significantly enhance the activity of ceftriaxone against MRSA in mice, suggesting it can be used as an antibiotic adjuvant to expand the antibiotic spectra of β-lactams to MRSA (41).

## DISCUSSION

In summary, we have discovered a new class of broad-spectrum antibacterial agents, trypyricins. They each possess two hydrophobic aromatic residues and several cationic functional groups, making them small-molecule mimics of the natural cationic AMPs. Mechanistic studies also showed that trypyricins directly target and disrupt bacterial cytoplasmic membranes. However, they are distinct from colistin, as the antibacterial activity of trypyricins is not dependent on the presence of lipopolysaccharide. Trypyricins also increase membrane fluidity as opposed to the anionic AMP, daptomycin, which decreases membrane fluidity. Compared to AMP drugs, trypyricins have the advantages of a broader spectrum of activity against both Gram-positive and Gram-negative bacteria, ease in synthesis and modification, and better *in vivo* stability. In addition, trypyricin 1 synergizes with β-lactam antibiotics against MRSA both *in vitro* and in a mouse model of thigh infection. Further studies are still required to evaluate the PK profile and efficacy of trypyricins as potential β-lactam adjuvant in human. Evolutionary studies showed that trypyricins, either alone or in combination with a β-lactam antibiotic, had extremely low potential for resistance development. These results indicated that trypyricins represent a new class of AMP-like small-molecule bacterial membrane-targeting agents and may be further developed as antibiotic adjuvants to fight against resistant bacteria in the clinic.

## MATERIALS AND METHODS

### Bacterial strains and growth media

*B. subtilis* strain NR-607, *E. faecium* strain HM-460, and *S. aureus* strains NRS384 and MRSA252 were obtained from BEI Resources. *S. aureus* strains 29213, BAA-1683, BAA-1717, BAA-1720, 43300, 33591, 33592, and 700789; *K. pneumoniae* strain 700603; *A. baumannii* strain BAA-1605; *P. aeruginosa* strain 27853; and *E. coli* strain 25922 were purchased from American Type Culture Collection, and *E. coli* strain CGSC 4213(ΔlptD) was obtained from Coli Genetic Stock Center. All strains were grown and maintained in Luria-Bertani (LB, Invitrogen) medium and agar unless otherwise mentioned, except for *Enterococcal* strains that were maintained in Bacto Brain Heart Infusion (VWR) medium and agar.

### Antimicrobial susceptibility testing

Broth microdilution experiments to determine MICs were carried out according to Clinical & Laboratory Standards Institute (CLSI) guidelines (17). Bacteria were grown to log phase in LB and diluted to OD_600_ 0.01 in Cation-Adjusted Mueller–Hinton Broth (CAMHB) media (Sigma-Aldrich) to inoculate into CytoOne clear, 96-well polystyrene untreated tissue culture plates (USA Scientific). Cation-adjusted Mueller–Hinton broth (CAMHB) (179 µL) was added to each well of the plate, followed by 1 µL of 200× compound in dimethyl sulfoxide (DMSO). Plates were then inoculated with 20 µL of diluted bacteria. This procedure was used to test the MIC in all strains except for enterococci, which were assayed in brain heart infusion (BHI) broth instead. The MIC was defined as the lowest compound concentration that produced a clear sample well after overnight (18 h) incubation. The MBC was determined by plating 10 µL of each clear well on a non-selective Mueller-Hinton (MH) agar plate.

### Checkerboard antimicrobial interaction assays and FICI determination

Checkerboard assays were set up and FICIs were determined as previously described using a variation on the conditions for standard CLSI antimicrobial susceptibility testing (42). For combination testing, each test agent was serially diluted at 8–10 different concentrations down and across a standard 96-well assay plate, respectively, to create an 8 × 10 matrix, where each well contained a different concentration combination of the test agents. Each matrix was set up in duplicate. The fractional inhibitory concentration (FIC) of each drug was calculated as the minimum concentration required to inhibit visible growth in the presence of the other test agent divided by the MIC of the drug. The FICI is the sum of the two FICs. A drug combination is considered synergistic where the FICI is ≤0.5.

### Time-kill kinetics assay

The mode of antibacterial growth inhibition of trypyricins was tested using a standard time-kill assay (43). Overnight cultures of bacteria (MRSA252 or *E. coli* ATCC 25922) were diluted to 10^6^ CFU/mL in CAMHB media. Two hundred microliters of diluted inoculum was added to each well of the plate. Trypyricins were diluted in DMSO and added to the test wells so that the final concentration of DMSO was 0.5% and trypyricins were tested at 0, 0.5× MIC, 1.0× MIC, 2.0× MIC, and 4.0× MIC. Aliquots of 10 µL were taken from each well at times 0, 2, 4, 6, and 24 h. Up to 12 10-fold dilutions were made of each aliquot, and 10 µL of each dilution was plated on non-selective LB agar plates. All plates were incubated at 35°C overnight, and CFU was counted for each dilution. The lower limit of detection was 100 CFU/mL. Bactericidal activity was defined as a 3 log_10_ CFU/mL reduction in bacterial density from starting inoculum (i.e., 99.9% killing). A graph of CFU per milliliter was plotted against time for all concentrations of each compound.

### Resistance selection

Resistant mutant evolution by serial passage was conducted similarly to previous reports (20). Serial dilution plates containing trypyricin 1, trypyricin 2, trypyricin 1 or 2 in combination with cefazolin (1:8 ratio), or a daptomycin control were prepared similarly to MIC plates and inoculated with 10^6^ CFU MRSA252. The plate was incubated for 24 h at 37°C, and the OD_600_ was measured using a BioTeK Epoch plate reader. Readings higher than 0.10 (background subtracted) were considered growth. For each treatment, an aliquot was taken from the highest concentration of treatment that allowed growth (i.e., 0.5 MIC), diluted 1:200, and passaged into a plate containing the same treatments. This process was repeated every day for 48 days. A glycerol stock (40% vol/vol) of the initial inoculum was stored and glycerol stocks of each treatment were stored whenever a change in MIC was observed and at the end of the experiment.

### Genomic DNA extraction, sequencing, and analysis

DNA was isolated from bacterial samples stored on day 1 and day 48. Isolation was carried out using an EZNA Bacterial Genomic DNA kit (Omega Biotech) following pre-treatment with lysostaphin (20 U/mL) at 37°C for 1 h. A Quant-iT PicoGreen dsDNA assay kit (Thermo Fisher) was used to quantify the isolated DNA. Library preparation was carried out using a NexteraXT DNA library preparation kit, and 2× 150 paired-end sequencing was performed using an Illumina MiSeq. These services were provided by Admera Health, LLC (South Plainfield, NJ). Sequence alignment, variant calling, and annotation were performed using the *breseq* 0.35.0 pipeline with the published MRSA252 genome as the reference (GenBank assembly accession: GCA_000011505.1) (21). Single nucleotide polymorphisms (SNPs) that were different from the day 1 and day 48 DMSO samples were further investigated.

### Macromolecular radiolabel incorporation assay

The macromolecular radiolabel incorporation assay was carried out as previously described by ImQuest Life Sciences (Fredrick, MD) (27). *S. aureus* NRS384 grown to OD_600_ 0.3–0.5 was diluted to OD_600_ 0.15. For the macromolecular incorporation assay, 2× concentrations of each test compound to be used were prepared in 50 µL in a 96-well plate. To the compound preparations, 50 µL of bacteria in a medium containing the appropriate radiolabeled precursors (acetic acid, 1–2-^14^C sodium salt–fatty acid synthesis; thymidine, 2-^14^C–DNA synthesis; uridine, 2-^14^C–RNA synthesis; *N*-acetyl glucosamine, glucosamine 1-^14^C–cell wall biosynthesis; and L-amino acid mixture, ^14^C–protein biosynthesis) was added. The mixture was incubated for 20 min. At this point, the mixture was transferred to a fresh plate containing 100 µL of pre-chilled 10% trichloroacetic acid (TCA). The plate was incubated on ice for 1 h for precipitation of radio precursor-incorporated material. Next, all contents in the 96-well microtiter plate were transferred to a filter plate, and any non-precipitated material was removed by vacuum filtration. The filter plate was washed three times with 200 µL of cold 5% TCA and three times with 200 µL of cold 75% ethanol. The filters were transferred to a MicroBeta cassette, and 25–50 µL of scintillation fluid was added to each well. Finally, the plate was sealed and counted on a MicroBeta Scintillation counter. Counts were normalized to the untreated control to calculate the percent incorporation of radio-labeled precursors. Positive controls were triclosan (fatty acid synthesis), ciprofloxacin (DNA synthesis), rifampin (RNA synthesis), vancomycin (cell wall synthesis), and chloramphenicol (protein synthesis).

### Fluorescence microscopy-based cytological profiling

Phenotypic evaluation in *E. coli* CGSC 4213 (ΔlptD) was carried out as previously described (30). From an overnight culture, cells were diluted 1:100 in fresh media and grown to exponential phase (OD_600_ ~0.3). The culture was divided and treated with 5× MIC of each compound for 30 min at 35–37°C. Sub-MIC treatment morphology experiments using *B. subtilis* NR-607 were performed similarly to previous reports (44). Cells from an overnight culture were diluted 1:100 and grown to mid-log phase. These were divided and treated with ½ MIC of daptomycin, trypyricin 1, or trypyricin 2. Treated cells were allowed to grow at 37°C for 2 hr. After treatment, cultures were harvested by centrifugation at 3,000 g for 30 s and stained in 1/10 vol of staining solution (2 µg/mL FM 4–64, 2 µg/mL DAPI, and 0.5 µM SYTOX Green in phosphate-buffered saline [PBS]). SYTOX Green was not included in the stain for the sub-MIC treated *B. subtilis*.

For imaging, 5–10 µL of stained cells were transferred to an agarose pad prepared on a slide (10% LB, 1% agarose) and covered with a poly-lysine-treated coverslip. Bacterial cells were imaged using an Olympus IX81 inverted wide-field microscope equipped with appropriate filters for imaging Texas Red (560/620), DAPI (350/460), and FITC (470/525), and a ×100 UPLSAPO super apochromat objective with 1.4 numerical aperture (Olympus). For each image, four optical sections were taken 0.33 µM apart, and exposure time for each channel was consistent throughout the experiment. Image planes were deconvoluted in ImageJ (National Institutes of Health) using the Diffraction-PSF-3D and iterative deconvolution plugin, and the maximum z-stack projection is shown.

### SYTOX Green membrane permeability assay

SYTOX Green (Thermo Fisher) accumulation was used to measure cytoplasmic membrane disruption as previously described (26, 44). *B. subtilis* NR-607 or *E. coli* ATCC 25922 were grown to early-mid-exponential phase in LB media (OD_600_ 0.2–0.4). Cells were collected by centrifugation, washed with PBS, and resuspended in PBS at an OD_600_ of 0.2. SYTOX Green was added to the cells in PBS to a final concentration of 1 µM. After the mixture was incubated for 30 min at room temperature, 100 µL of each mixture was transferred to opaque black half-area 96-well plates (Costar 3694), and baseline SYTOX Green signal was measured for 5 min on a PerkinElmer 2102 EnVision Multilabel plate reader (Ex 485/Em 525). Following the baseline measurement, 1 µL of DMSO or 100× compounds in DMSO was added (final concentration of DMSO in the assay was 1%). Triton X-100 (0.5%) was used as a positive control to achieve total cell lysis (45).

### Cytoplasmic membrane fluidity assay

Laurdan dye was used to assess membrane fluidity as previously described using *B. subtilis* NR-607 (46). Bacteria were grown overnight in LB and sub-cultured 1:100 in LB-supplemented 1.25-mM CaCl_2_, 0.5-mM MgCl_2_, and 0.2% glucose. The bacteria were grown to mid-log phase (OD_600_ 0.40) and stained with 10-µM Laurdan dye (in 1% DMSO) for 5 min. Cells were washed three to four times in pre-warmed PBS supplemented with 1.25-mM CaCl_2_, 0.5-mM MgCl_2_, and 0.2% glucose. The cell suspension was diluted 1:1 in supplemented PBS and added to opaque black half-area 96-well plates (100 µL per well, Costar 3694), and a baseline measurement was taken over 5 min using a Perkin Elmer 2102 EnVision Multilabel plate reader (Ex 340/Em 440 and 510 nm) at room temperature. Compound in DMSO (1% final DMSO concentration) was added to the plate. DMSO alone was added as a negative control, and benzyl alcohol (1%) was added as a positive control. The Laurdan signal was measured continuously for 5 min. General polarization of the emission signal was calculated using the following formula:


$$GP=\frac{\left(I_{440}\;-\;I_{510)}\right)}{\left(I_{440}\;+\;I_{510)}\right)}$$

where *I*_440_ is the fluorescence emission intensity at 440 nm, and *I*_510_ is the fluorescence emission intensity at 510 nm. The GP increases from baseline as the membrane rigidity increases and decreases as the fluidity increases.

### Liposome leakage assay

Carboxy-fluorescein-loaded liposomes were prepared and assayed as described previously (38, 47). A phospholipid mixture selected to mimic the *S. aureus* membrane was used (50% phosphoglycerol and 50% cardiolipin), and all acyl chains were unsaturated (18:1) for ease of use (39). To assay the effect of compound treatment on phospholipid membrane integrity, 10 µM (1:500 dilution) of *S. aureus* mimicking liposomes were challenged with dilutions of trypyricins, daptomycin, or cefazolin for 20 min. Liposome damage was measured as the percentage of fluorescein leakage relative to complete lysis with 0.15% Triton X-100 and was calculated as follows:


$$\%Lysis=\left(\frac{F_{Treatment}-F_{DMSO}}{F_{Triton}-F_{DMSO}}\right)\times 100$$

where *F* is the fluorescein signal, and the max signal is obtained after treatment with 0.15% Triton X-100. The liposome lysis assay was prepared in black half-area 96-well plates blocked with 5% BSA. Fluorescent signal was read using an EnVision multilabel plate reader equipped with appropriate filters.

### Mammalian cytotoxicity assay

HEK293 (ATCC CLR-1573) or HepG2 (ATCC HB-8065) cells were cultured in Dulbecco’s modified Eagle’s medium supplemented with 10% fetal calf serum and 1% penicillin/streptomycin. Cells were seeded in a white, cell culture-treated, 96-well plate (Corning 3917) and were incubated at 37°C in 5% CO_2_/95% air for 24 h before the medium was removed from each well and replaced with 99.5 µL of warmed fresh medium. Test compound (0.5 µL) in twofold serial dilution in DMSO was added to each well. Each series was performed in triplicate. After incubation at 37°C for 48 h, the plates were equilibrated to room temperature for 30 min. One hundred microliters of Cell Titer-Glo reagent (Promega) was added to each well and mixed for 2 min in an orbital shaker. The plate was incubated at room temperature for another 10 min before the luminescence of each well was recorded with an Envision Multilabel Plate Reader (PerkinElmer).

### Hemolytic assay

The working solutions of test compound were prepared at 25 mM and 12.5 mM in DMSO. Two microliters of test compound in working solution or 100 µL of 5% Triton X-100 (diluted in saline, a positive control) was added into a new tube. Nine hundred ninety-eight microliters or 900 µL human red blood cells were added into the tube, and the samples were vortexed gently for 5 s and equilibrated at 25°C for 2 min. Normal saline was used as a negative control. Sample solutions were washed four times with 5 mL of normal saline, and the supernatant was discarded after 5 min of centrifugation. An aliquot of 4 mL of sterile water was added to the remaining plug and then vortexed thoroughly to destroy all red blood cells. All samples were centrifuged for 4 min at 3,000 rpm. One hundred microliters of the supernatant was transferred to a new plate and diluted with 900 µL water. After being vortexed, 200 µL of each sample was transferred to a new plate, and absorbance at 545 nm was recorded. The percentage of hemolysis was calculated as follows:

*% Hemolysis* = (A_neg_ – A_test_)/(A_neg_) × 100

where A_*neg*_ is the absorbance of the negative control, and A_test_ is absorbance of the test sample.

### Pharmacokinetic analysis

Pharmacokinetics analysis for trypyricins and ceftriaxone was performed by Pharmaron (Beijing, China). Six- to eight-week-old male CD-1 mice were administered the test agent at 2 mpk via i.v. or 10 mpk via i.p. in a triturated formulation with 5% DMSO and 5% cremophor in a 20% HP-*β*-CD solution in saline. Blood samples were collected at 5, 15, and 30 min, as well as 1, 2, 4, 6, 8, and 24 h post-dose, with three replicates per time point. At each time point, 30-µL blood was collected and transferred into plastic microcentrifuge tubes containing anticoagulant of Heparin-Na, mixed, and centrifuged at 4,000 *g* for 5 min at 4°C to obtain the plasma. Liquid chromatography−tandem mass spectrometry was performed on an AB Sciex 6,500 (trypyricin 1) or 5,500 (trypyricin 2) Triple-Quad liquid chromatography with tandem mass spectrometry (LC/MS/MS) instrument.

Chromatographic separation was achieved using a HALO 160-Å ES-C18, 2.7-µM 2.1 × 50 mm column (trypyricin 1) or a Waters XSELECT CSH C18 2.5-µM 2.1 × 50 mm column (trypyricin 2). Mobile phase A consisted of 5% acetonitrile in water with 0.1% vol/vol formic acid in water. Mobile phase B consisted of 95% acetonitrile in water with 0.1% vol/vol formic acid in water. For trypyricin 1, the analyte was eluted with a gradient of 5%–95% mobile phase B at a flow rate of 0.5 mL/min with 5-µL injection volume. For trypyricin 2, the analyte was eluted with a gradient of 0%–95% mobile phase B at a flow rate of 0.6 mL/min with 20-µL injection volume. Electrospray ionization was performed in positive mode. Transition of *m*/*z* 635.16 → 498.10 was used to identify trypyricin 1; transition of *m*/*z* 568.16 → 278.10 was used to identify trypyricin 2; and transition of *m*/*z* 393.40 → 372.30 was used to identify dexamethasone as an internal standard. Concentration of test samples was interpolated from a standard curve derived from the intensity values of standards (1–1,000 ng/mL).

### Neutropenic mouse thigh infection model

These studies were conducted by NeoSome Life Sciences (Lexington, MA) (48). All procedures were performed to NeoSome IACUC policies and guidelines as well as OLAW standards. Female CD-1 mice (Charles River Laboratories) were allowed to acclimate for 5 days prior to start of study. Mice received two doses of cyclophosphamide on days −4 and −1 with 150 and 100 mpk delivered i.p., respectively. MRSA strain ATCC 43300 was prepared for infection from an overnight plate culture. A portion of the plate was resuspended in sterile saline and adjusted to an optical density (OD) of 0.10 at 625 nm. The adjusted bacterial suspension was further diluted to target an infecting inoculum of 1.0 × 10^5^ CFU per mouse; the actual inoculum size was 8.3 × 10^4^ CFU per mouse. Mice were inoculated with 100 µL of the prepared bacterial suspension via intramuscular injection into the right rear thigh. Test agents were formulated in 5% DMSO, 5% polysorbate 80, and 90% of a 20% HP-*β*-CD solution prepared in saline prior to the first dose. Vancomycin was prepared in sterile deionized (DI) water. Beginning at 1 h post-infection, mice were dosed with either test agent or vancomycin. Mice receiving test agents were delivered intraperitoneally at 10 mL/kg. For combination treatment groups, test agents were formulated in combination and delivered as a single injection for each dose schedule. Vancomycin was delivered through subcutaneous injection (10 mL/kg). Five animals were dosed per group. One group was euthanized at initiation of therapy (T = 1 h) and CFUs were determined. All remaining mice were euthanized at 25 h post-infection. At termination, thighs were aseptically excised, weighed, and homogenized to a uniform consistency in 2 mL of sterile saline. The homogenates were serially diluted and plated on bacterial growth media. The CFUs were enumerated after overnight incubation. The average and standard deviations for each group were determined. For the infection model using MRSA strain ATCC BAA-1717, the actual inoculum size was determined as 8.3 × 10^4^ CFU per mouse, and four animals were dosed per group.

## SUPPLEMENTAL MATERIAL

Supplementary Information aac.00051-23-s0001.pdf synthesis, figures, and tables

ASM does not own the copyrights to Supplemental Material that may be linked to, or accessed through, an article. The authors have granted ASM a non-exclusive, world-wide license to publish the Supplemental Material files. Please contact the corresponding author directly for reuse.
