# Supplementary material for: A small-molecule membrane fluidizer re-sensitizes methicillin-resistant Staphylococcus aureus (MRSA) to β-lactam antibiotics
Source: Antimicrob Agents Chemother. 2023 Sep 8;67(10):e00051-23. doi: 10.1128/aac.00051-23 (PMC10583677; doi:10.1128/aac.00051-23)
Supplement: Supplementary Information — synthesis, figures, and tables [file aac.00051-23-s0001.pdf]

Supporting Information for

A Small-Molecule Membrane Fluidizer Re-sensitizes Methicillin-Resistant *Staphylococcus aureus* (MRSA) to *β*-Lactam Antibiotics

Jessica D. Podoll, Emma Rosen, Wei Wang, Yuefeng Gao, Jing Zhang,* Xiang Wang*

*Corresponding authors: Jing Zhang and Xiang Wang.
Email: zhang@recreopharm.com; xiangw@colorado.edu

This PDF file includes:

Supporting text
Figures S1 to S6
Tables S1 to S4

Supporting Information Text

Materials and Methods

Synthesis of trypyricins. The synthetic work was conducted by Medicilon (Shanghai, China). All common solvents and chemicals were used as purchased without further purification. The progress of all reactions was monitored on Aldrich precoated silica gel plates (with fluorescence indicator UV254) using ethyl acetate/*n*-hexane or methanol/dichloromethane as solvent system, and by a Waters HPLC-MS. High resolution mass spectrometry analysis of trypyricins was conducted using a Waters Synapt G2 mass spectrometry. Column chromatography was performed with Aldrich silica gel 60 (230−400 mesh ASTM) with the solvent mixtures specified in the corresponding experiment. Spots were visualized by irradiation with ultraviolet light at 254 nm. Purity of all final compounds was 98% or higher according to HPLC analysis. ^{1}H and ^{19}F NMR spectra were recorded on Bruker Avance 400 MHz using solvents as indicated in the experimental section. ^{13}C-NMR spectra were recorded on 75 MHz using solvents as indicated in the experimental section. Chemical shifts are given in parts per million (ppm) (δ relative to residual solvent peak for ^{1}H). ^{1}H shifts were referenced to $CDCl_3$ at 7.26 ppm and ^{13}C shifts were referenced to DMSO-d_6 at 39.51 ppm.

1-(2,2-Diethoxyethyl)-1H-imidazole (1)

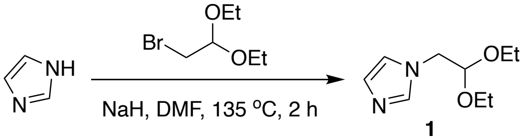

To a solution of NaH (14.1 g, 0.37 mol) in DMF (200 mL) was added 1H-imidazole (25 g, 0.37 mol) at 0 °C. The mixture was stirred at 0 °C for 1 h, then 2-bromo-1,1-diethoxyethane (72 g, 0.37 mol) was added and stirred at 135 °C for 1 h. The mixture was cooled to room temperature and quenched by saturated NH_4Cl solution. The residue was extracted with 3x 500 mL ethyl acetate and then washed by 2x 300 mL water. The organic phase was dried over $MgSO_4$, filtered, and concentrated to afford the title compound **1** (60 g, 75% yield) as a yellow oil. ^{1}H-NMR (400 MHz, DMSO-d_6): δ 7.59 (s, 1H), 7.15 (t, *J* = 1.1 Hz, 1H), 6.87 (s, 1H), 4.66 (t, *J* = 5.3 Hz, 1H), 4.04 (d, *J* = 5.3 Hz, 2H), 3.58–3.70 (m, 2H), 3.37–3.44 (m, 2H), 1.07 (t, *J* = 7.0 Hz, 6H) ppm. LCMS $[M+H]^+$ = 185.2.

1-((1H-imidazol-1-yl)methyl)-6-chloro-2,3,4,9-tetrahydro-1H-pyrido[3,4-b]indole (3)

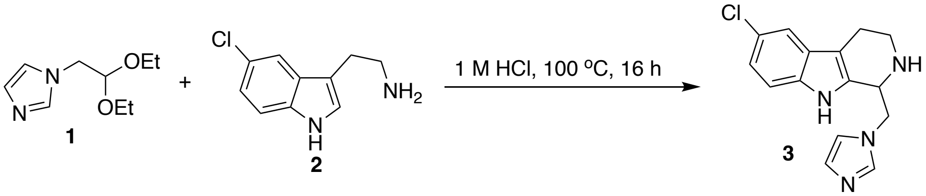

To a solution of acetal **1** (20 g, 108 mmol) in 1 N HCl (150 mL) was added 5-chlorotryptamine **2** (25 g, 108 mmol). The mixture was stirred at 100 °C for 16 h. After cooled to 0 °C, the residue was treated with saturated NaOH solution until the pH >9. The resulting precipitates were collected and washed with water and MeOH to afford the title compound **3** (18 g, 58% yield) as a yellow solid. ^{1}H-NMR (400 MHz, DMSO-d_6): δ 11.12 (s, 1H), 7.56 (s, 1H), 7.42 (d, *J* = 2.0 Hz, 1H), 7.37 (d, *J* = 8.5 Hz, 1H), 7.09 (s, 1H), 7.05 (dd, *J* = 8.6, 2.1 Hz, 1H), 6.85 (s, 1H), 4.34–4.45 (m, 1H), 4.17–4.27 (m, 2H), 2.94–3.03 (m, 1H), 2.83–2.92 (m, 1H), 2.52 (t, *J* = 5.6 Hz, 2H), 2.41 (s, 1H) ppm. LCMS: $[M+H]^+$ = 287.1.

***Tert*-butyl 4-(5-chloro-6-(trifluoromethyl)pyridin-2-yl)-3-(hydroxymethyl) piperazine-1-carboxylate (6)**

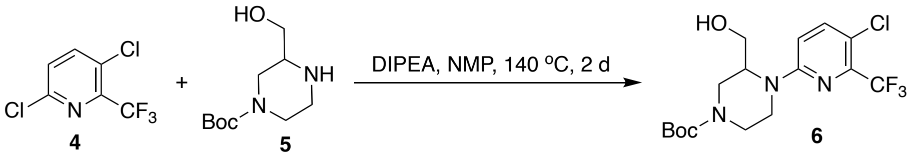

A mixture of 3,6-dichloro-2-(trifluoromethyl)pyridine **4** (50 g, 232 mmol), *tert*-butyl 3-(hydroxy-methyl)piperazine-1-carboxylate **5** (75 g, 347 mmol), and DIPEA (120 g, 930 mmol) in anhydrous NMP was stirred at 140 °C for 2 days. The mixture was cooled to room temperature, poured into 500 mL water, and extracted with 2x 500 mL ethyl acetate. The combined organic phase was washed with 8x 500 mL water to remove NMP. The organic phase was then washed with brine, dried over Na_2SO_4, filtered, and concentrated *in vacuo*. Purification by silica gel chromatography using ethyl acetate in petroleum ether (0–30%) afforded the title compound **6** (40 g, 43% yield) as a yellow solid. ^{1}H-NMR (400 MHz, DMSO-d_6): δ 7.81 (d, *J* = 9.1 Hz, 1H), 7.11 (d, *J* = 9.2 Hz, 1H), 4.81 (t, *J* = 5.1 Hz, 1H), 4.24 (s, 1H), 4.15 (d, *J* = 12.8 Hz, 1H), 4.01–4.08 (m, 1H), 3.86 (s, 1H), 3.40–3.53 (m, 2H), 2.86–3.16 (m, 3H), 1.42 (s, 9H) ppm. LCMS $[M+H]^+$ = 396.0.

***Tert*-butyl 4-(5-chloro-6-(trifluoromethyl)pyridin-2-yl)-3-formylpiperazine-1-carboxylate (7)**

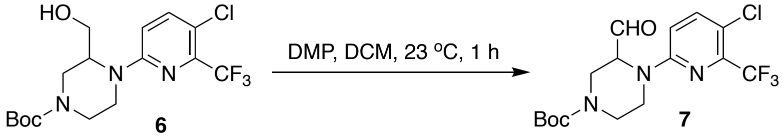

To a solution of alcohol **6** (43 g, 108 mmol) in anhydrous dichloromethane (400 mL) was added Dess-Martin periodinane (55 g, 130 mmol) at 0 °C. The mixture was stirred at room temperature for 1 h before concentrated. Purification by silica gel column chromatography using ethyl acetate in petroleum ether (0–20%) afforded aldehyde **7** (30 g, 70% yield) as a yellow solid. ^{1}H-NMR (400 MHz, DMSO-d_6): δ 9.63 (s, 1H), 7.92 (d, *J* = 9.1 Hz, 1H), 7.20 (d, *J* = 9.1 Hz, 1H), 5.10 (d, *J* = 2.9 Hz, 1H), 4.51 (d, *J* = 13.9 Hz, 1H), 3.99 (d, *J* = 13.0 Hz, 1H), 3.82 (d, *J* = 11.6 Hz, 1H), 3.24–3.33 (m, 1H), 2.99–3.21 (m, 2H), 1.40 (s, 9H) ppm.

***Tert*-butyl 3-((1-((1H-imidazol-1-yl)methyl)-6-chloro-1,3,4,9-tetrahydro-2H-pyrido[3,4-b] indol-2-yl)methyl)-4-(5-chloro-6-(trifluoromethyl)pyridin-2-yl)piperazine-1-carboxylate (8)**

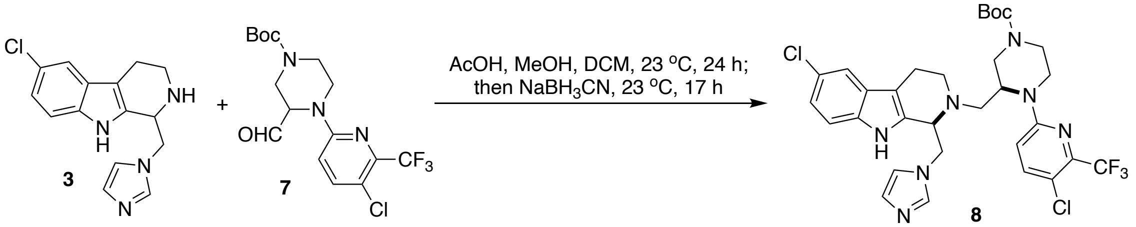

To a solution of the aldehyde **7** (24 g, 62.7 mmol) and amine **3** (12 g, 41.8 mmol) in anhydrous DCM/MeOH (60 mL/80 mL) was added AcOH (2 mL). The mixture was stirred at 25 °C for 24 h, and then the mixture was cooled to 0 °C. AcOH (2 mL) was added followed by the addition of $NaBH_3CN$ (7.9 g, 125 mmol). The mixture was stirred at 25 ℃ for 17 h. The solvent was then removed, and the residue diluted with ethyl acetate and water. Saturated $NaHCO_3$ solution was next added at 0 °C until pH was 8~9. The organic phase was washed with water, brine, dried over sodium sulfate, filtered, and concentrated. Purification by silica gel column chromatography using MeOH in DCM (0–10%) or by C18 reverse phase column chromatography using CH_3CN/0.1% TFA in H_2O (0–60%) to give a light yellow solid. The solid was recrystallized with ethyl acetate/hexane or CH_3CN/H_2O to give the title compound **8** (5.4 g, 20% yield) as a white solid. ^{1}H-NMR (400 MHz, DMSO-d_6): δ 10.96 (s, 1H), 7.81 (d, *J* = 9.2 Hz, 1H), 7.40 (s, 1H), 7.37 (d, *J* = 2.0 Hz, 1H), 7.33 (d, *J* = 8.6 Hz, 1H), 7.03 (dd, *J* = 8.6, 2.1 Hz, 2H), 6.89 (s, 1H), 6.78 (s, 1H), 4.43 (s, 1H), 4.32 (d, J = 5.8 Hz, 2H), 3.80–4.05 (m, 4H), 2.88–3.11 (m, 4H), 2.54–2.80 (m, 4H), 2.28 (d, *J* = 15.5 Hz, 1H), 1.39 (s, 9H) ppm. LCMS $[M+H]^+$ = 664.3.

The single crystal of compound **8** chloroform solvate was obtained from liquid vapor diffusion experiment using chloroform as the solvent. The relative stereochemical configuration of **8** was

determined by X-ray analysis. The structure has been deposited in CCDC (deposition number: 2155094).

3-((2-((4-(*Tert*-butoxycarbonyl)-1-(5-chloro-6-(trifluoromethyl)pyridin-2-yl)piperazin-2-yl)methyl)-6-chloro-2,3,4,9-tetrahydro-1H-pyrido[3,4-b]indol-1-yl)methyl)-1-((1-(*tert*-butoxycarbonyl)azetidin-3-yl)methyl)-1H-imidazol-3-ium chloride (10)

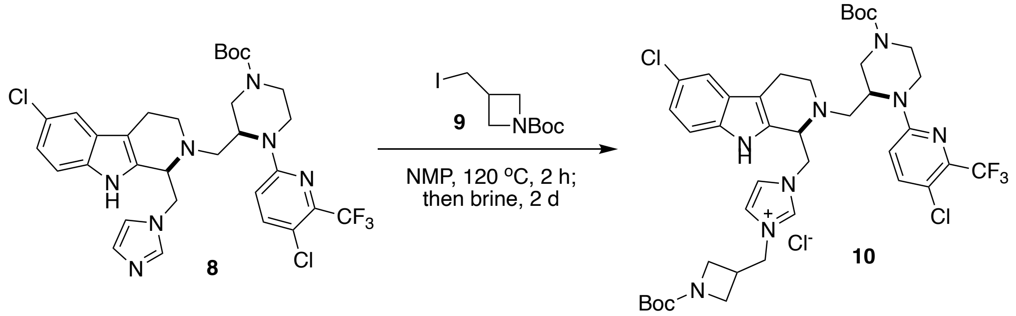

A solution of the imidazole **8** (6.00 g, 9.04 mmol) and iodide **9** (26.0 g, 90.4 mmol) in anhydrous NMP (70 mL) was stirred at 120 °C for 2 h. The mixture was cooled to room temperature before dichloromethane (300 mL) was added. The resulting solution was washed with 6x 300 mL water to remove NMP, dried, filtered, and concentrated. Purification by silica gel chromatography using 0–10% MeOH in DCM gave a yellow solid. The solid was dissolved in a mixture solution (DCM/MeOH/brine=1:1:2) and stirred at room temperature for 2 days (salt exchange from iodide to chloride). The organic layer was dried, filtered, concentrated and purified by silica gel column eluting with 0–10% MeOH in DCM to give the title compound **10** (5.0 g, 64% yield) as a white solid. LCMS $[M]^+$ = 833.6.

1-(Azetidin-3-ylmethyl)-3-((6-chloro-2-((1-(5-chloro-6-(trifluoromethyl)pyridin-2-yl)piperazin-2-yl)methyl)-2,3,4,9-tetrahydro-1H-pyrido[3,4-b]indol-1-yl)methyl)-1H-imidazol-3-ium chloride (trypyricin 1)

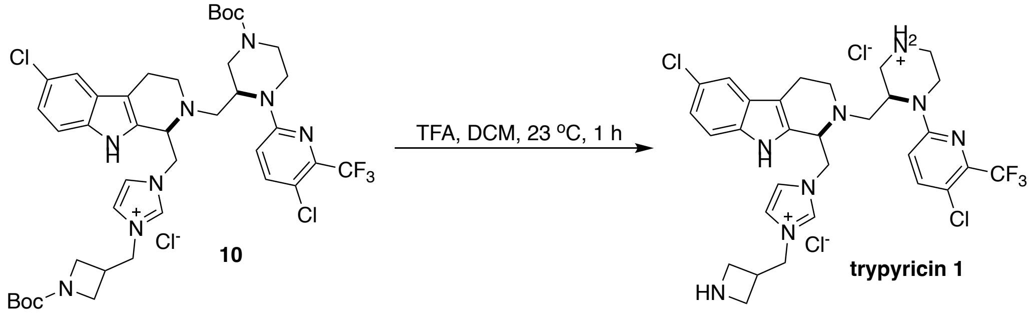

To a solution of 3-((2-((4-(*tert*-butoxycarbonyl)-1-(5-chloro-6-(trifluoromethyl)pyridin-2-yl)piperazin-2-yl)methyl)-6-chloro-2,3,4,9-tetrahydro-1H-pyrido[3,4-b]indol-1-yl)methyl)-1-((1-(*tert*-butoxycarbonyl)azetidin-3-yl)methyl)-1H-imidazol-3-ium chloride **10** (4.0 g, 4.6 mmol) in anhydrous dichloromethane (20 mL) was added TFA (10 mL) at 0 °C. The mixture was stirred at room temperature for 1 h before concentrated *in vacuo.* The residue was purified by C18 reverse column chromatography eluting with CH_3CN/0.1%HCl in H_2O (0–40%) to give trypyricin 1 (2.4 g, 70% yield) as a light yellow solid. ^{1}H-NMR (400 MHz, DMSO-d_6): δ 11.42 (s, 1H), 10.15 (d, *J* = 9.1 Hz, 1H), 9.78 (s, 1H), 9.33 (s, 3H), 7.87–7.95 (m, 2H), 7.79 (s, 1H), 7.39 (s, 1H), 7.36 (d, *J* = 8.6 Hz, 1H), 7.16 (d, *J* = 9.2 Hz, 1H), 7.05 (dd, *J* = 8.6, 2.0 Hz, 1H), 4.78 (s, 1H), 4.62–4.72 (m, 3H), 4.40–4.61 (m, 2H), 4.01–4.17 (m, 3H), 3.86–3.98 (m, 2H), 3.39–3.52 (m, 1H), 3.09–3.33 (m, 5H), 2.86–3.03 (m, 3H), 2.70–2.83 (m, 1H), 2.42 (d, *J* = 15.1 Hz, 1H) ppm. ^{19}F-NMR (400 MHz, DMSO-d_6): δ –64.84 (s) ppm. ^{13}C-NMR (75 MHz, DMSO-d_6): δ 154.5, 141.6, 140.0 (q, *J* = 33.0 Hz, CF_3), 137.5, 134.7, 129.7, 127.2, 123.6, 122.9 (2C), 122.6, 121.8, 117.7, 116.9, 113.1 (3C), 107.4, 57.7, 50.2, 49.7, 47.9, 47.8, 47.7, 46.5, 45.1, 41.4, 37.2, 31.7, 16.7 ppm. LCMS: $[M]^+$ = 633.0. HRMS *m/z* calcd. for $C_{30}H_{34}Cl_2F_3N_8$: $[M]^+$ 633.2236, found 633.2243.

2-Azido-1,1-diethoxyethane (11)

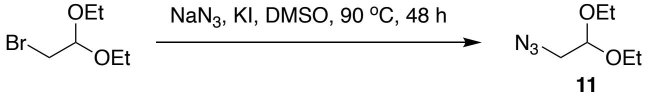

A mixture of NaN_3 (2.5 g, 38 mmol), KI (0.50 g, 30 mmol) and 2-bromo-1,1-diethoxyethane (5.0 g, 26 mmol) in 20 mL DMSO was stirred at 90 °C for 48 h. The reaction mixture was then poured into water (100 mL) and extracted with 3x 50 mL ethyl acetate. The combined organic layer was dried over Na_2SO_4 and concentrated *in vacuo* to afford the title compound **11** (2.4 g, 58% yield) as a colorless oil. ^{1}H-NMR (400 MHz, $CDCl_3$): δ 4.60 (t, *J* = 5.3 Hz, 1H), 3.55–3.77 (m, 4 H), 3.24 (d, *J* = 5.2 Hz, 2 H), 1.24 (t, 6 H).

1-(Azidomethyl)-6-chloro-2,3,4,9-tetrahydro-1H-pyrido[3,4-b]indole (12)

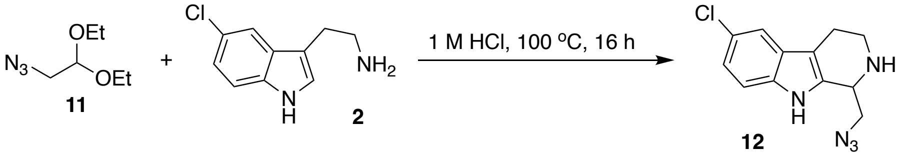

A mixture of acetal **11** (1.9 g, 12 mmol), 5-chlorotryptamine **2** (1.8 g, 8 mmol) and TFA (1.8 g, 16 mmol) in butanol (40 mL)/H_2O (3 mL) was stirred at 100 °C in a sealed tube for 16 h. The reaction mixture was purified by C18 reverse phase column chromatography to afford tryptoline **12** (1.3 g, 42% yield) as a brown solid. ^{1}H-NMR (400 MHz, DMSO-*d*$_6$): δ 10.96 (s, 1H), 7.42 (s, 1H), 7.31 (d, *J* = 8.4 Hz, 1H), 7.02 (dd, *J* = 8.4 Hz, 2.4 Hz, 1H), 4.17 (s, 1H), 3.54–3.66 (m, 2H), 3.07–3.12 (m, 1H), 2.90–2.94 (m, 1H), 2.55–2.59 (m, 3 H) ppm. LCMS: $[M+H]^+$ = 262.0.

***Tert*-butyl3-((1-(azidomethyl)-6-chloro-1,3,4,9-tetrahydro-2H-pyrido[3,4-b]indol-2-yl)-methyl)-4-(5-chloro-6-(trifluoromethyl)pyridin-2-yl)piperazine-1-carboxylate (13)**

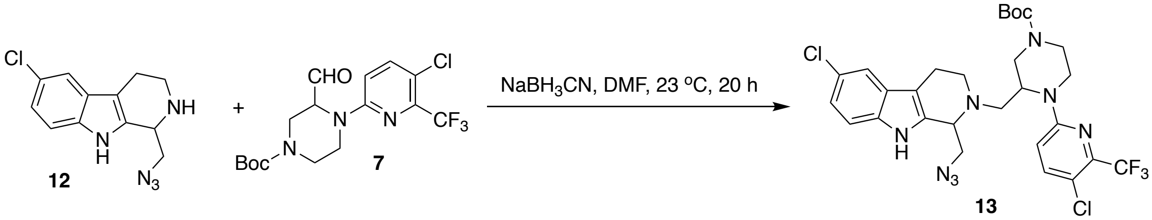

To a solution of the aldehyde **7** (1.0 g, 2.5 mmol) in anhydrous DMF (10 mL) was added the amine **12** (670 mg, 2.5 mmol). The resulting mixture was stirred at 40 °C for 14 h before $NaBH_3CN$ (488 mg, 7.5 mmol) was added. The reaction was stirred at room temperature for 30 min. The mixture was then concentrated and purified by silica gel column chromatography (petroleum ether:EtOAc = 8:1) to afford the title compound **13** (1.1 g, 68% yield) as a yellow solid. LCMS: $[M+H]^+$ = 639.2

***Tert*-butyl3-((1-(aminomethyl)-6-chloro-1,3,4,9-tetrahydro-2H-pyrido[3,4-b]indol-2-yl)me-thyl)-4-(5-chloro-6-(trifluoromethyl)pyridin-2-yl)piperazine-1-carboxylate (14)**

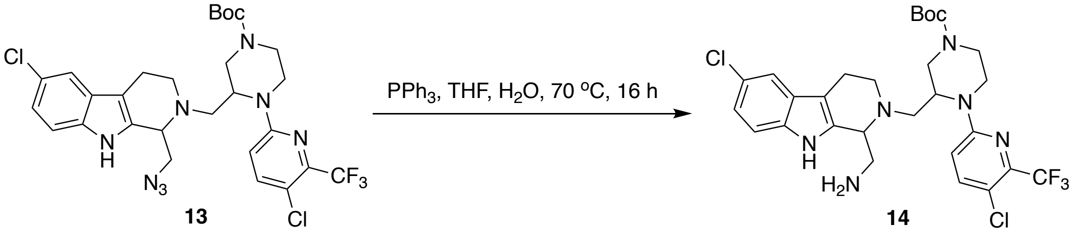

To a solution of azide **13** (1.1 g, 1.7 mmol) in THF/H_2O (5 mL/5 mL) was added triphenylphosphine (670 mg, 2.6 mmol). The mixture was stirred at 70 °C for 16 h. The reaction mixture was then cooled to room temperature, concentrated, and extracted with 3x 50 mL ethyl acetate. The combined organic layer was dried over Na_2SO_4, filtered, and concentrated *in vacuo*. Purification via silica gel column chromatography (petroleum ether: ethyl acetate = 40:1) afforded the title compound **14** (1.0 g, 70% yield) as a yellow solid. ^{1}H-NMR (400 MHz, CD_3OD): *δ* 7.76 (d, *J* = 8.8

Hz, 1H), 7.42 (s, 1 H), 7.31 (d, J = 8.8 Hz ,1H), 7.18 (d, J = 8.4 Hz, 1H), 7.09 (d, J = 8.4 Hz, 1H), 4.93–5.04 (m, 2 H), 4.24–4.28 (m, 2 H), 3.83–3.88 (m, 1 H), 3.13–3.63 (m, 8 H), 2.86–3.03 (m, 3 H), 1.39 (s, 9H). LCMS: $[M+H]^+$ = 613.3.

***Tert*-butyl 3-((1-(((1-(tert-butoxycarbonyl)azetidin-3-yl)amino)methyl)-6-chloro-3,4-dihydro-1H-pyrido[3,4-b]indol-2(9H)-yl)methyl)-4-(5-chloro-6-(trifluoromethyl)pyridin-2-yl)piperazine-1-carboxylate (16)**

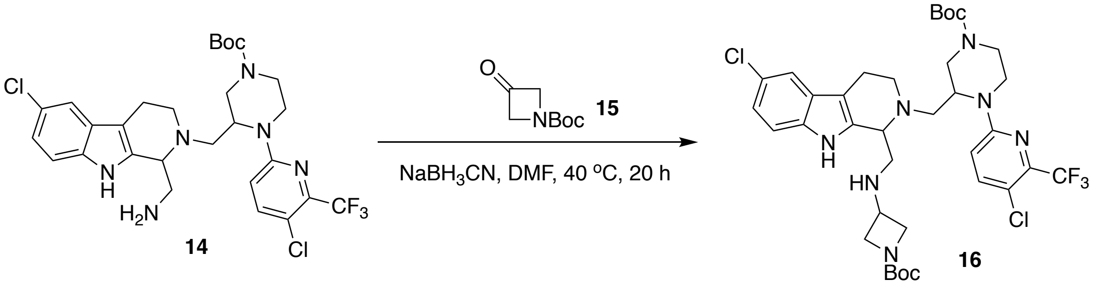

A mixture of the amine **14** (500 mg, 0.80 mmol), *tert*-butyl 3-oxoazetidine-1-carboxylate **15** (412 mg, 2.4 mmol), and AcOH (0.5 mL) in anhydrous DMF (10 mL) was stirred at 40 °C for 16 h before $NaBH_3CN$ (272 mg, 4.0 mmol) and AcOH (0.5 mL) was added and stirred at 40 °C for another 4 h. The solution was quenched by saturated $NaHCO_3$ at 0 °C, diluted with 60 mL EtOAc, and then washed with H_2O and brine. The organic phase was dried over Na_2SO_4, filtered, and concentrated *in vacuo*. The residue was purified by silica gel column chromatography eluting with MeOH in DCM (0–5%) to afford the title compound **16** (480 mg, 77% yield) as a yellow solid. LCMS $[M+H]^+$ = 782.2.

***N*-((6-Chloro-2-((1-(5-chloro-6-(trifluoromethyl)pyridin-2-yl)piperazin-2-yl)methyl)-2,3,4,9-tetrahydro-1H-pyrido[3,4-b]indol-1-yl)methyl)azetidin-3-amine (trypyricin 2)**

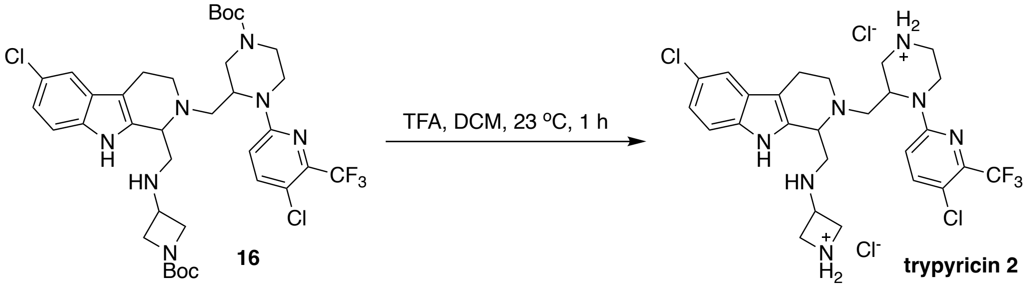

To a solution of compound **16** (260 mg,0.33mmol) in dichloromethane (2 mL) was added TFA (2 mL). The mixture was stirred at room temperature for 1 h. The mixture was then concentrated and purified using reverse phase Prep-HPLC to give trypyricin 2 (109 mg, 47% yield) as a light yellow solid. ^{1}H-NMR (400 MHz, CD_3OD): δ 7.78 (d, J = 9.2 Hz, 1H), 7.43 (d, J =2.0 Hz, 1H), 7.28–7.32 (m, 2H), 6.84 (dd, J =8.4, 2.0 Hz, 1H), 5.34 (brs, 1H), 4.53–4.56 (m, 1H), 4.45–4.46 (m, 1H), 4.32–4.40 (m, 4H), 4.00–4.04 (m, 1H), 3.36–3.57 (m, 9H), 3.21–3.25 (m, 2H), 3.00–3.07 (m, 1H), 2.74–2.78 (m, 1H). LCMS: $[M+H]^+$ = 568.1. HRMS m/z calcd. for $C_{26}H_{31}Cl_2F_3N_7$: $[M+H]^+$ 568.1970, found 568.1979.

***Tert*-butyl (*E*)-3-((1-((2,3-bis(*tert*-butoxycarbonyl)guanidino)methyl)-6-chloro-1,3,4,9-tetrahydro-2H-pyrido[3,4-b]indol-2-yl)methyl)-4-(5-chloro-6-(trifluoromethyl)pyridin-2-yl)piperazine-1-carboxylate (17)**

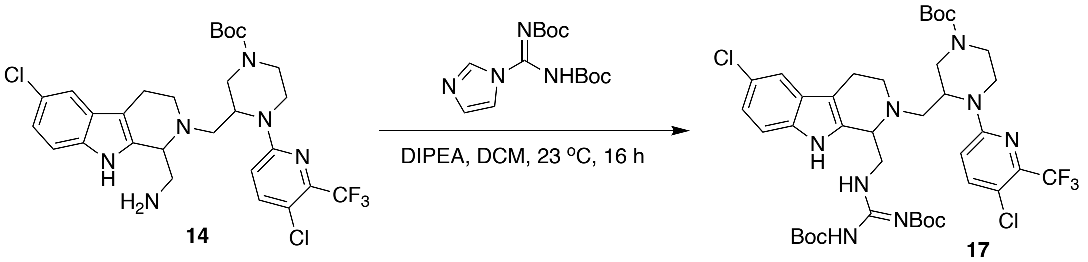

To a solution of amine **14** (500.0 mg, 0.8 mmol) in anhydrous DCM (10 mL) was added diisopropylethylamine (210.0 mg, 1.6 mmol) and *tert*-butyl (*E*)-(((*tert*-butoxycarbonyl)imino) (1H-imidazol-1-yl)methyl)carbamate (301.0 mg, 1.0 mmol). The mixture was stirred at room temperature for 16 h and concentrated to afford the title compound **17** (700.0 mg, 99% yield) as a white solid. The crude product was taken to the next step without further purification. LCMS $[M+H]^+$ = 855.2

1-((6-Chloro-2-((1-(5-chloro-6-(trifluoromethyl)pyridin-2-yl)piperazin-2-yl)methyl)-2,3,4,9-tetrahydro-1H-pyrido[3,4-b]indol-1-yl)methyl)guanidine (trypyricin 3)

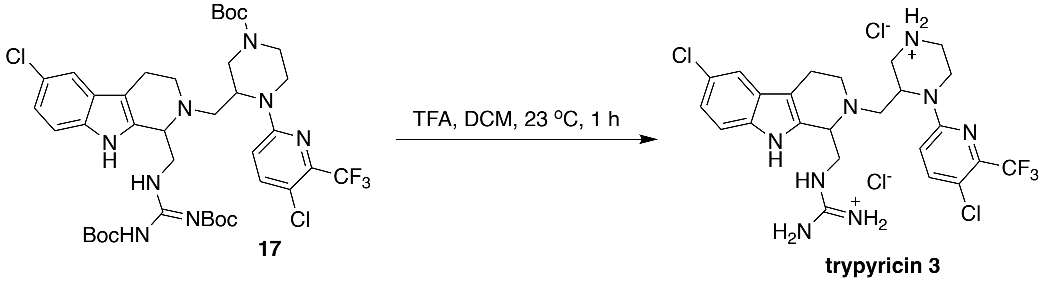

To a solution of compound **17** (1.6 g, 1.8 mmol) in anhydrous DCM (15 mL) was added TFA (5 mL). The mixture was stirred at room temperature for 3 h. The reaction mixture was then concentrated and purified directly using a reverse phase prep-HPLC to afford trypyricin 3 (450.0 mg, 43% yield). ^{1}H-NMR (400 MHz, CD_3OD): δ 7.71 (d, *J* = 8.0 Hz, 1H), 7.37 (s, 1H), 7.26 (d, *J* = 8.6 Hz, 1H), 7.02–7.08 (m, 2 H), 4.12–4.28 (m, 2H), 3.87 (d, *J* = 13.1 Hz, 1H), 3.47–3.62 (m, 3H), 3.30–3.40 (m, 4H), 3.15–3.23 (m, 2H), 3.03–3.09 (m, 1H), 2.73–2.89 (m, 2H), 2.50–2.53 (m, 1H). LCMS $[M+H]^+$ = 555.2. HRMS *m/z* calcd. for $C_{24}H_{28}Cl_2F_3N_8$: $[M+H]^+$ 555.1766, found 555.1778.

1-(2-Amino-2-oxoethyl)-3-((2-((4-(*tert*-butoxycarbonyl)-1-(5-chloro-6-(trifluoromethyl)-pyridin-2-yl)piperazin-2-yl)methyl)-6-chloro-2,3,4,9-tetrahydro-1H-pyrido[3,4-b]indol-1-yl)methyl)-1H-imidazol-3-ium bromide (18)

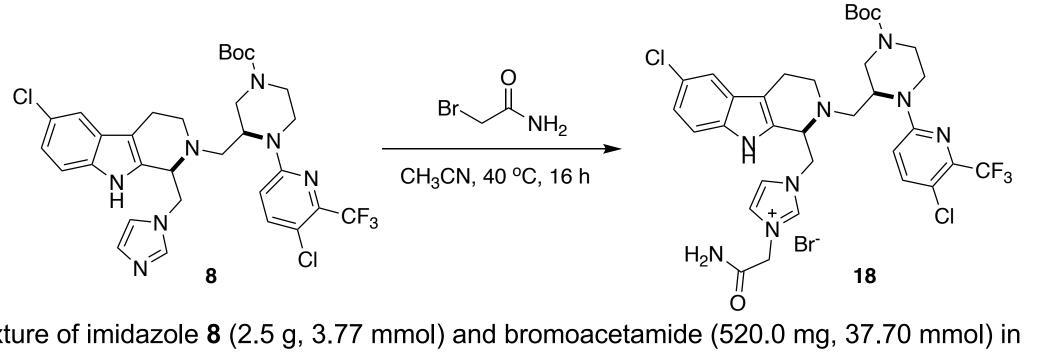

A mixture of imidazole **8** (2.5 g, 3.77 mmol) and bromoacetamide (520.0 mg, 37.70 mmol) in anhydrous CH_3CN was stirred at 40 °C for 16 h. The reaction mixture was then cooled to room temperature, concentrated, and purified by silica gel column chromatography (DCM:MeOH = 10:1) to afford the title compound **18** (946.0 mg, 33% yield) as a yellow solid. LCMS $[M]^+$ = 721.2.

1-(2-Amino-2-oxoethyl)-3-((6-chloro-2-((1-(5-chloro-6-(trifluoromethyl)pyridine-2-yl)pipe-ra-zin-2-yl)methyl)-2,3,4,9-tetrahydro-1H-pyrido[3,4-b]indol-1-yl)methyl)-1H-imidazol-3-ium chloride (trypyricin 4)

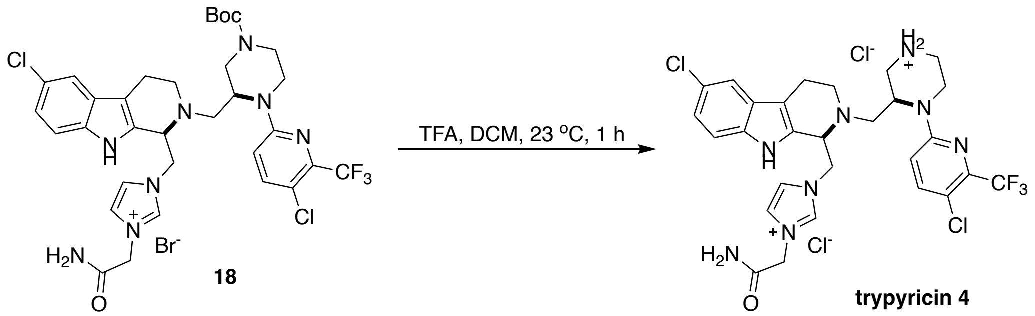

18

trypyricin 4

To a solution of compound **18** (946 mg, 1.25 mmol) in anhydrous DCM (10 mL) was added TFA (2 mL). The reaction mixture was stirred at room temperature for 2 h. The reaction mixture was then concentrated and purified by reverse phase prep-HPLC to afford trypyricin 4 (320 mg, 39% yield) as a yellow solid. ^{1}H-NMR (400 MHz, DMSO-d_6): δ 11.30 (s, 1H), 9.48 (s, 2H), 9.21 (s, 1 H), 7.90 (d, J = 9.1 Hz, 1 H), 7.83 (s, 1 H), 7.73 (s, 1 H), 7.37 (d, J = 8.6 Hz, 1 H), 7.15 (d, J = 9.1 Hz, 2H), 7.07 (d, J = 2.0 Hz, 1H), 7.05 (d, J = 2.0 Hz, 1H), 5.08–5.19 (m, 2H), 4.64–4.73 (m, 2H), 4.44–4.56 (m, 2H), 4.08 (d, J = 13.6 Hz, 1H), 3.07–3.27 (m, 3H), 2.92–3.00 (m, 2H), 2.77–2.85 (m, 2H), 2.71–2.71 (m, 2H), 2.39–2.67 (m, 2H). LCMS $[M]^+$ = 621.9. HRMS *m/z* calcd. for $C_{28}H_{30}Cl_2F_3N_8O$: $[M]^+$ 621.1872, found 621.1874.

Figures and Tables

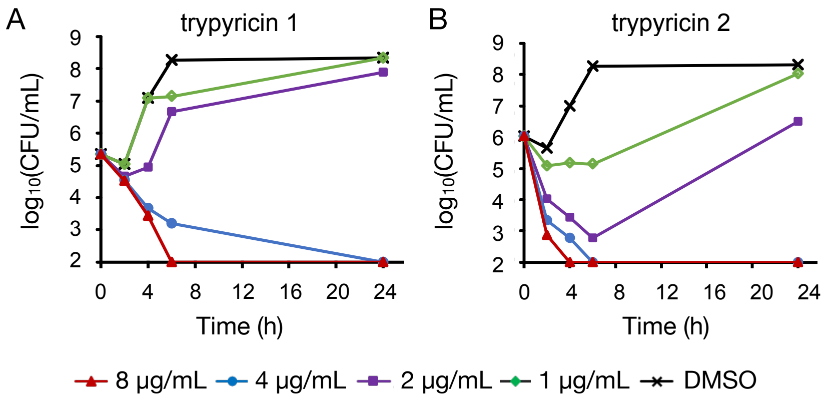

Fig. S1. Time-kill kinetic curves of trypyricins 1 (A) and 2 (B) against *E. coli* ATCC 25922.

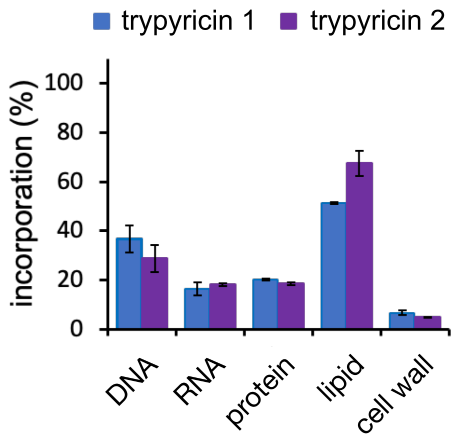

Fig. S2. Trypyricins 1 and 2 inhibit all five major biosynthetic pathways in *S. aureus* NRS384 at 4x MIC.

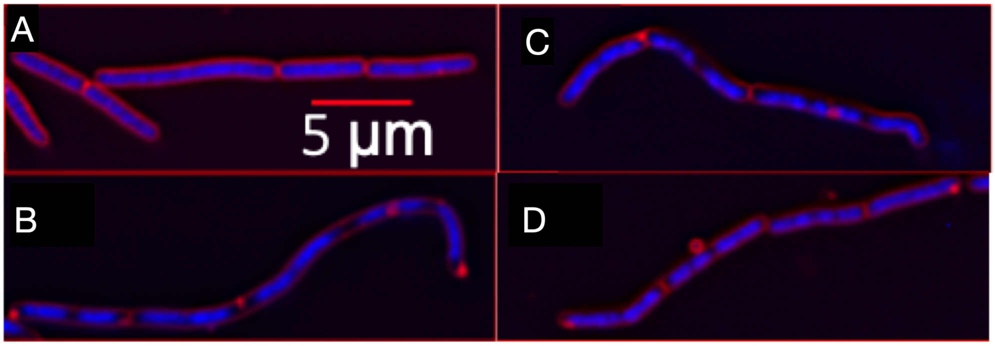

Fig. S3. Fluorescence-based cytological profiling of *B. subtilis treated* with DMSO, daptomycin, or trypyricins. *B. subtilis* cells were grown in the presence of DMSO (A), daptomycin (B), trypyricin 1 (C), or trypyricin 2 (D), respectively, for 2 hours before stained with membrane dye FM 4-64 (red) and DAPI (blue).

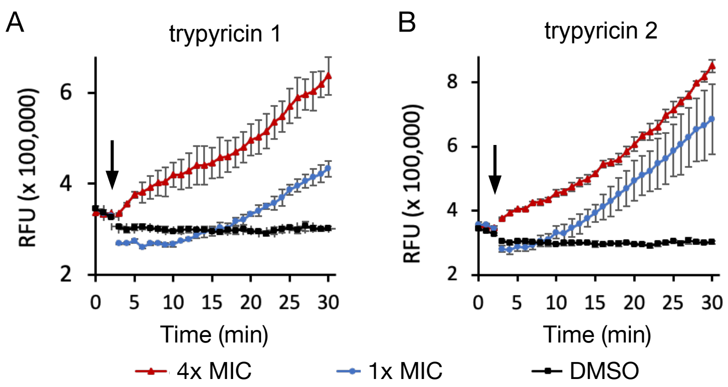

Fig. S4. CYTOX Green uptake assay showed trypyricins 1 (A) and 2 (B) disrupted *E. coli* membrane integrity.

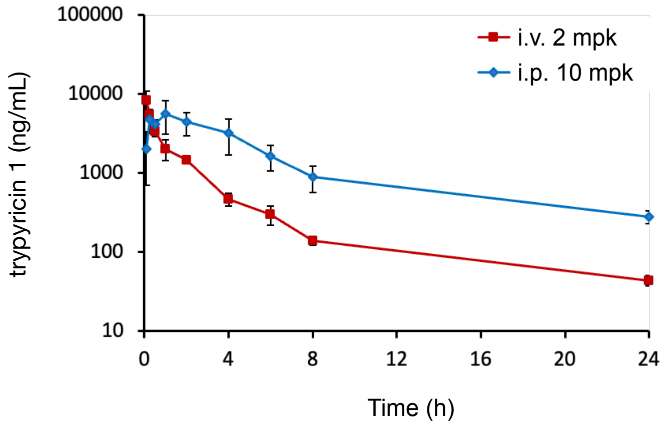

Fig. S5. Pharmacokinetic analysis of trypyricin 1. The mean plasma concentrations of try-ricin 1 after a single i.v. injection of 2 mpk or a single i.p. injection of 10 mpk of trypyricin 1 (3 CD-1 mice for each route of administration). Data are the mean of plasma concentrations, and the error bars represent the standard deviation from 3 animals at each time point. Pharmacokinetic parameters were calculated and listed in Extended Data Table 3.

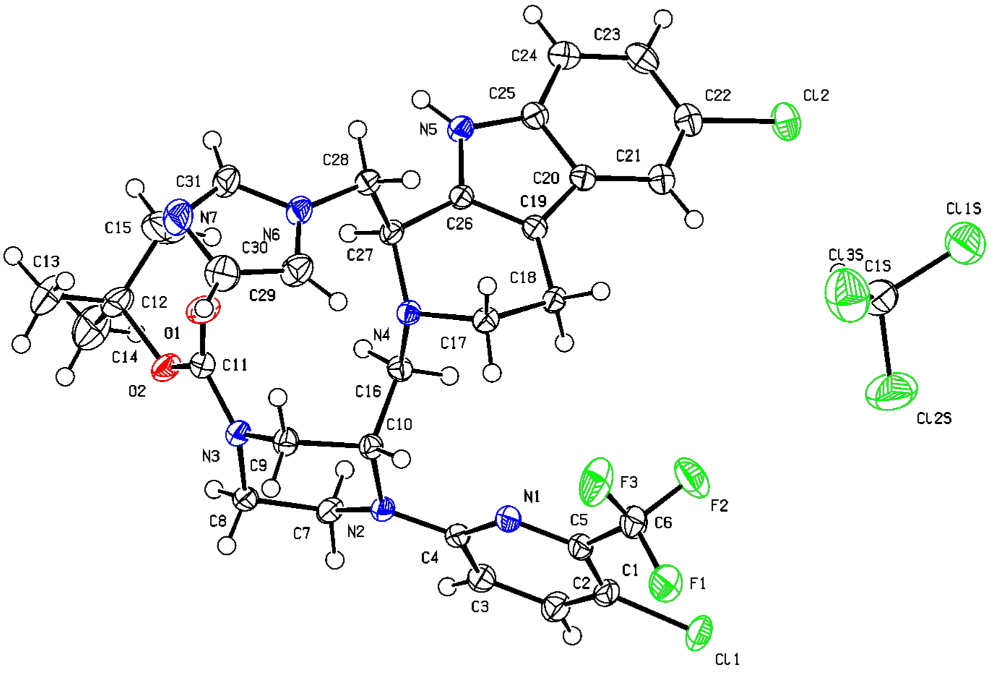

Figure S6. Ellipsoid plot of intermediate **8** chloroform solvate.

Table S1. Antibacterial activity of trypyricins and other antibiotics.[a]

Species and strain	MIC (μg/mL)							
	trypyricins				Cfz	Ctx	Dap	Col
	1	2	3	4				
B. subtilis NR-607	0.5	0.5	0.5	1	0.25	0.5	2	16
S. aureus ATCC 29213 (MSSA)	0.5	0.5	0.5	0.5	0.25	0.5	2	>64
E. coli ATCC 25922	4	2	2	4	2	0.031	-	1
E. faecium HM-460	1	1	1	1	>64	>64	>16	-
K. pneumoniae ATCC 700603	8	4	8	4	>64	16	-	1
A. baumannii ATCC BAA-1605	4	2	2	4	>64	>64	-	0.5
P. aeruginosa ATCC 27853	4	2	4	4	>64	>64	-	1
E. cloacae ATCC BAA-2468	4	2	2	4	>64	>64	-	0.5
S. aureus BAA-1717 (MRSA)	0.5	0.5	0.5	0.5	>64	>64	2	-
S. aureus MRSA252 (MRSA)[b]	0.5(1)	0.5(2)	0.5(2)	0.5(4)	>64	>64	2	-
S. aureus BAA-1720 (MRSA)	0.5	0.5	1	0.5	>64	>64	2	-
S. aureus BAA-1683 (MRSA)	1	1	1	1	>64	>64	1	-
S. aureus 33592 (MRSA)	0.5	0.5	1	0.5	>64	>64	2	-
S. aureus NRS384 (MRSA)	0.5	0.5	0.5	0.5	32	16	1	-
S. aureus 33591 (MRSA)	1	1	1	1	>64	16	2	-
S. aureus 433000 (MRSA)	0.5	0.5	0.5	0.5	16	64	2	-
S. aureus 700789 (MRSA)	0.5	0.5	0.5	0.5	16	32	2	-

[a] MICs were determined in cation-adjusted Muller Hinton Broth 2 (CAMHB) media.

[b] Numbers in parenthesis are MICs in the presence of 4% human serum albumin. Cfz: cefazolin; Ctx: ceftriaxone; Dap: daptomycin; Col: colistin.

Table S2. Amino acid changes located in open reading frames after *in vitro* selection.

Treatment	Mutated gene	%population with mutation	Function	Base change	Amino acid change
trypyricin 1 _replicate 1	glpD	100	glycerol-3-phosphate dehydrogenase/ oxidase	Δ1 bp G (94)	Ala 32 frame shift
trypyricin 1 _replicate 1	SAR_RS11940	100	DeoR/GlpR transcriptional regulator	G334 > A	Gly112 > Arg
trypyricin 1 _replicate 2	accB	100	acetyl-CoA carboxylase biotin carboxyl carrier protein	A32 > G	Glu11 > Gly
trypyricin 1 _replicate 2	dat	52	D-amino-acid transaminase	T17 > A	Leu6 > Stop
trypyricin 2 _replicate 1	ahpC	100	peroxiredoxin	G98 > A	Gly33> Asp
trypyricin 2 _replicate 1	mvk	100	mevalonate kinase	C598 >T	Pro99 > Leu
trypyricin 2 _replicate 1	SAR_RS05505	100	YktB family protein		Asp192 > Stop
trypyricin 2 _replicate 1	accC	94	acetyl-CoA carboxylase biotin carboxylase subunit	A590> G	Tyr197> Cys
trypyricin 2 _replicate 2	no mutations recovered				
cefazolin/trypyricin 1_replicate 1	accC	100	acetyl-CoA carboxylase biotin carboxylase subunit	A902 >G	Glu301 > Gly
cefazolin/trypyricin 1_replicate 1	SAR_RS14435	100	capsular polysaccharide biosynthesis protein CapA	C248 > T	Pro83 > Leu
cefazolin/trypyricin 1_replicate 2	SAR_RS12680	100	galactose mutarotase	Δ3 bp AAT (541-543)	ΔAsn 181
cefazolin/trypyricin 1_replicate 2	SAR_RS14440	100	TetR/AcrR family transcriptional regulator	+T 137	Ser 46 Frame Shift
cefazolin/trypyricin 2_replicate 1	SAR_RS05080	74	CPBP family intramembrane metalloprotease	+T 450	Ser 150 Frame Shift
cefazolin/trypyricin 2_replicate 1	SAR_RS11225	92	PP2C family protein-serine/threonine phosphatase	G464 > T	Gly155 > Val (GGA→GTA)
cefazolin/trypyricin 2_replicate 1	SAR_RS11295	90	response regulator transcription factor	G335 > A	Arg 112 > Gln

cefazolin/trypyricin 2_replicate 2	glpK	83	glycerol kinase GlpK	+T 1147	Leu 383 Frame Shift
cefazolin/trypyricin 2_replicate 2	glpK	83	glycerol kinase GlpK	+A 1147	Leu 383 Frame Shift
cefazolin/trypyricin 2_replicate 2	glpD	100	glycerol-3-phosphate dehydrogenase/ oxidase	C1512 > A	Tyr504 > Stop
daptomycin	mprF	100	bifunctional lysylphosphatidylglycerol flippase/synthetase MprF	C287 > T	Ala96 > Val
daptomycin	SAR_RS03445	100	ABC transporter ATP-binding protein	C220 >T	Leu74 > Phe
daptomycin	SAR_RS03460	100	inorganic phosphate transporter	Δ6 bp CTAGAG (110-115)	Thr 37 Frame Shift
daptomycin	SAR_RS10970	100	TrkH family potassium uptake protein	C19 > A	Pro7 > Thr

Table S3. Pharmacokinetic profiles of trypyricins and ceftriaxone in mice.[a]

	i.v. at 2 mpk					i.p. at 10 mpk			
Compound	C_{max} (ng/mL)	$t_{1/2}$ (h)	V_{dss} (L/kg)	Cl (mL/min/kg)	DNAUC (mg·h/mL)	C_{max} (ng/mL)	$t_{1/2}$ (h)	DNAUC (mg·h/mL)	F (%)
trypyricin 1	10445	7.56	0.81	3.02	5357	6360	7.56	3366	63
trypyricin 2	7862	21.2	5.11	7.47	1866	2483	8.90	1195	64
ceftriaxone	3581	0.93	0.22	3.65	4564	1795	0.95	4575	100

[a]Compounds were administered at 2 mpk body weight intravenously or 10 mpk body weight intraperitoneally to CD1 mice. Three animals were used for each compound. All values are means of three biological replicates. Plasma samples were analyzed by LC–MS/MS. C_{max} = maximal plasma concentration. $t_{1/2}$ = half-life. V_{dss} = volumes of distribution calculated by the steady-state method. Cl = clearance. DNAUC = dose-normalized area under the curve. F = bioavailability.

Table S4. Crystal data and structure refinement of intermediate 8 chloroform solvate (CCDC 2155094).

**Sample ID**	8103114-05-A4
**Empirical formula**	$C_{31}H_{34}Cl_2F_3N_7O_2 \cdot CHCl_3$
**Formula weight**	783.92
**Temperature**	120.00(10) K
**Wavelength**	Cu/K_α (λ = 1.54184 Å)
**Crystal system, space group**	Orthorhombic, $P2_12_12_1$
**Unit cell dimensions**	a = 10.93830(10) Å<br>b = 10.95210(10) Å<br>c = 30.3370(2) Å<br>α = 90º<br>β = 90º<br>γ = 90º
**Volume**	3634.29(5) $Å^3$
***Z*, Calculated density**	4, 1.433 g/cm^3
**Absorption coefficient**	4.118 mm^{-1}
***F*(000)**	1616.0
**Crystal size**	0.077 × 0.057 × 0.056 mm^3
**2 Theta range for data collection**	5.826 to 152.288
**Limiting indices**	$-13 \leq h \leq 13$<br>$-13 \leq k \leq 13$<br>$-37 \leq l \leq 36$
**Reflections collected/Independent reflections**	41731/6927 [R_{int}=0.0325, R_{sigma}=0.0166]
**Refinement method**	Full-matrix least-squares on F^2
**Completeness**	96.50 %
**Data / restraints / parameters**	6927/0/445
**Goodness-of-fit on F^2**	1.027
**Final R indices [I ≥ 2sigma(I)]**	R_1 = 0.0338, wR_2 = 0.0905
**Final R indices [all data]**	R_1 = 0.0344, wR_2 = 0.0910
**Largest diff. peak and hole**	0.48/-0.55 e.$Å^{-3}$
**Flack parameter**	0.001(4)
**Bayesian statistics on Bijvoet differences[1]**	Hooft y = 0.000(1), P2(true)=1.000,<br>P3(true) = 1.000, P3(rac-twin) = 0.0E+00,<br>P3(false) = 0.0E+00
